# Nanoisland SERS-Substrates for Specific Detection and Quantification of Influenza A Virus

**DOI:** 10.3390/bios14010020

**Published:** 2023-12-29

**Authors:** Gleb Zhdanov, Alexandra Gambaryan, Assel Akhmetova, Igor Yaminsky, Vladimir Kukushkin, Elena Zavyalova

**Affiliations:** 1 Chemistry Department, Lomonosov Moscow State University, 119991 Moscow, Russia; gleb.zhdanov@chemistry.msu.ru (G.Z.); zlenka2006@gmail.com (E.Z.); 2 Moscow Institute of Physics and Technology, Institute of Quantum Technologies, 141700 Dolgoprudny, Russia; 3 Chumakov Federal Scientific Centre for Research and Development of Immune and Biological Products RAS, 108819 Moscow, Russia; 4 Physics Department, Lomonosov Moscow State University, 119991 Moscow, Russia; assel1505@yandex.ru (A.A.); yaminsky@nanoscopy.ru (I.Y.); 5 Osipyan Institute of Solid State Physics of the Russian Academy of Science, 142432 Chernogolovka, Russia; kukushvi@mail.ru

**Keywords:** aptamer, aptasensor, influenza, nanoisland, SERS

## Abstract

Surface-enhanced Raman spectroscopy (SERS)-based aptasensors for virus determination have attracted a lot of interest recently. This approach provides both specificity due to an aptamer component and a low limit of detection due to signal enhancement by a SERS substrate. The most successful SERS-based aptasensors have a limit of detection (LoD) of 10–100 viral particles per mL (VP/mL) that is advantageous compared to polymerase chain reactions. These characteristics of the sensors require the use of complex substrates. Previously, we described silver nanoisland SERS-substrate with a reproducible and uniform surface, demonstrating high potency for industrial production and a suboptimal LoD of 4 × 10^5^ VP/mL of influenza A virus. Here we describe a study of the sensor morphology, revealing an unexpected mechanism of signal enhancement through the distortion of the nanoisland layer. A novel modification of the aptasensor was proposed with chromium-enhanced adhesion of silver nanoparticles to the surface as well as elimination of the buffer-dependent distortion-triggering steps. As a result, the LoD of the Influenza A virus was decreased to 190 VP/mL, placing the nanoisland SERS-based aptasensors in the rank of the most powerful sensors for viral detection.

## 1. Introduction

The recent pandemic of COVID-19 (CoronaVirus Disease of 2019) caused by SARS-CoV-2 (Severe Acute Respiratory Syndrome-related CoronaVirus 2), along with common outbreaks of influenza A virus raises the question of rapid diagnostics in crowded places like airports, city transport, clinics, universities, etc. Current acceptable techniques do not solve the problem of rapid diagnostics having either a high limit of detection (LoD) of 10^6^–10^8^ viral particles per mL (VP/mL) along with a 15 min time of analysis in the case of Lateral Flow Immunoassay (LFIA) [1,2] or an excellent LoD of 10^2^–10^3^ VP/mL along with a 2 h time of analysis in the case of polymerase chain reaction with reverse transcription (RT PCR) [3,4,5]. The compromised solutions have higher LoDs with a characteristic time of analysis of about 1 h; e.g., reverse transcription loop-mediated isothermal amplification (RT LAMP) has a LoD of 10^4^ VP/mL and a time of analysis of 1 h [6,7].

Nucleic acid aptamers (hereafter called simply aptamers) are artificially structured oligonucleotides that are able to bind a specific target with high affinity. Aptamers are chemically synthesized with a wide variety of modifications, e.g., labels or anchors. These properties make aptamers excellent recognition elements for advanced applications [8,9]. Aptamers have been selected for a wide variety of viruses, including SARS-CoV-2 [10,11], influenza A [12,13], human immunodeficiency virus [14,15], hepatitis virus [16,17,18], etc. Aptamer-based sensors (aptasensors) have been developed for a variety of targets with incredibly low limits of detection (LoD) and good analytical ranges of determination. Several reviews are recommended to compare different approaches and analytical techniques for aptamer-based detection of viruses [19,20].

Surface-enhanced Raman spectroscopy (SERS) is one of the most robust techniques that can be easily combined with aptamer-based recognition. SERS provides 10^6^–10^8^-fold amplification of the signal, allowing the detection of single-bound molecules. Several works on virus determination with SERS-based aptasensors in biological fluids have been published recently. Here we discuss the SERS-based aptasensors with LoD values compared to or lower than the LoD of polymerase chain reaction with reverse transcription (≤10^3^ VP/mL) with a time of analysis below 20 min. Interestingly, antibody-based SERS sensors concede to aptasensors in LoD values and time of analysis [21,22]; the possible reason is a large size of proteins as compared with aptamers, as well as the SERS decrease during protein adsorption, demanding a complex multilayer architecture of the sensors.

The lowest LoD was achieved using polymeric membranes with a silver nanoisland coating. The metal coating was functionalized with aptamers for influenza A virus (IAV) through the thiol group. The aptamers bound viruses during the filtration of influenza-containing biological fluid. The spectra of several virus particles cannot be detected directly. So, a Raman label was conjugated to the aptamer; the reorientation of the label during virus binding provided an analytical signal. The LoD of the aptasensor was as low as 10 VP/mL, with a time of analysis of 15 min [22]. Interestingly, the sandwich-like approach with two aptamers for catching and labeling the virus was also realized using polymeric membranes with silver nanoisland coatings with a much higher LoD of 2 × 10^4^ VP/mL [23]. Thus, binary complexes are more preferred than ternary ones for ultra-sensitive applications. In both cases, scanning electron microscopy revealed aggregates of metal nanoparticles after virus binding. Presumably, a virus distorts the surface due to its highly affine interaction with an aptamer. Polymeric membranes with silver nanoisland coatings are cheap to produce; however, sensors for multiplex detection have not been reported up to date.

The next SERS-based aptasensor with lithographic SERS substrates provided detection of SARS-CoV-2 with a LoD of 100 VP/mL, influenza A with a LoD of 600 VP/mL, and adenovirus with a LoD of 70 VP/mL [21] within 17 min. Au/Ag/Cr columns with a width of 310–1500 nm provided a highly reproducible analytical signal. The substrate was divided into four zones, each of which was functionalized with an individual aptamer with a conjugated Raman label. The multiplex sensor was able to decode mixtures of respiratory viruses. The characteristics of the aptasensor are optimal for practical implementation. However, the production of these substrates requires highly technological equipment, which is a single constraint of these aptasensors. 

One more SERS-based aptasensor with suitable characteristics used colloidal silver nanoparticles with membrane prefiltration. The LoD was 10^3^ VP/mL of IAV, and the time of analysis was 15 min [24]. Colloidal nanoparticles are cheap to produce and very stable during storage. The sole restriction of this aptasensor is the absence of the possibility of a multiplex analysis.

Here we describe the upgrade of our previous SERS-based aptasensor with a suboptimal LoD. Silver nanoislands on silica oxide substrate were functionalized with thiolated aptamers; the bound IAV was detected with labeling with a secondary aptamer conjugated with a Raman label. The LoD of the sensor was 4 × 10^5^ VP/mL within 12 min [25]. This type of SERS substrate is very convenient for production. The nanostructured surface was formed during the thermal spraying of silver on the silica oxide substrate. Several zones can be formed using a mask during spraying for further implementation in a multiplex analysis. The possible reason for a high LoD is a distortion in the nanostructured surface similar to that reported in references [22,23]. Recently, the production of silver nanoislands on silica oxide substrates was optimized to increase the adhesion of the nanoparticles [26]. Here we step-by-step optimize the setup of our aptasensor, decreasing the distortion of the surface and providing characteristics comparable to the best examples of the published SERS aptasensors.

## 2. Materials and Methods

### 2.1. Reagents

Inorganic salts (AppliChem GmbH, Darmstadt, Germany, and Sigma-Aldrich, St. Louis, MO, USA) and phosphate-buffered saline (PBS) tablets (Ecoservice, Saint Petersburg, Russia) were used. Glutaric aldehyde, NaN_3_, dithiothreitol (DTT), 6-mercaptohexanol, and spermidine were purchased from Helicon (Moscow, Russia).

The following DNA oligonucleotides were synthesized by Synthol (Moscow, Russia): RHA0385-SH, (SH-(CH_2_)_6_)-TTGGGGTTATTTTGGGAGGGCGGGGGTT; RHA0385-Cy3, (Cy3)-TTGGGGTTATTTTGGGAGGGCGGGGGTT; SH-RHA0385-Cy3, (SH-(CH_2_)_6_)-TTGGGGTTATTTTGGGAGGGCGGGGGTT-(Cy3); RBD-1C-SH, (SH-(CH_2_)_6_)-CAGCACCGACCTTGTGCTTTGGGAGTGCTGGTCCAAGGGCGTTAATGGACA. The aptamer RHA0385 was selected as hemagglutinin by Shiratori et al. [27] and successfully used for influenza A detection [21,22,23,24,25].

### 2.2. Aptamer Assembly

The aptamers were assembled using the following algorithm. Aptamers were prepared in 20 μM concentrations in the PBS buffer (pH 7.4 with 8 mM Na_2_HPO_4_, 1.5 mM KH_2_PO_4_, 140 mM NaCl, 20 mM KCl) or TrisK buffer (10 mM Tris-HCl pH 7.5, 150 mM KNO_3_). The solutions were heated at 95 °C for 5 min. Then, the solutions were assembled at room temperature. Then, the solutions were diluted with water, PBS, or TrisNa buffer (10 mM Tris-HCl pH 7.5, 140 mM NaNO_3_, 10 mM KNO_3_) according to the techniques used. All solutions were produced using ultrapure water produced by Millipore (Merck Millipore, Burlington, MA, USA).

### 2.3. Viruses

IAV of the H7N1 subtype (strain A/chicken/Rostock/45/1934, 6th passage), Newcastle Disease Virus (NDV, LaSota Vaccine Strain), and allantoic fluid were provided by the Chumakov Federal Scientific Center for Research and Development of Immune and Biological Products of the Russian Academy of Sciences. The allantoic cavity of 10-day-old embryonated SPF chicken eggs was used for virus replication. Chicken eggs were incubated at 37 °C, cooled at 4 °C 48 h post-infection, and harvested 16 h later. The addition of 0.05% (*v*/*v*) glutaric aldehyde and 0.03% (*w*/*v*) NaN_3_ was used to inactivate the viruses and preserve the biological fluids. The viruses were stored at +4 °C. The concentrations of the viruses were estimated using a Nanoparticle Tracking Assay according to the previous work [22]. The hemagglutination activity of the viruses was determined according to Killian [28]. Solutions of the viruses are diluted step-by-step in PBS. The aliquots of 50 μL of solutions were placed in a V-shaped 96-well microtiter plate. Then, the same volume of 0.5% chicken red blood cells in the PBS was added. The hemagglutination assay was conducted at 4 °C for 1 h. The hemagglutination titer was estimated as the maximal dilution of the virus that did not cause the appearance of the red dot in the well; this well was assumed to contain 1 HAU of the virus in the probe. IAV had a hemagglutination activity of 4000 HAU; NDV had a hemagglutination activity of 512 HAU.

### 2.4. SERS-Substrates Production

Thin metal layers were deposited onto the silicon substrate with a silica oxide layer with a thickness of 300 nm. The deposition was carried out with the vacuum spraying system NANO 38 (Kurt J. Lesker Company, Jefferson Hills, PA, USA) equipped with an automatic thickness controller. A spraying rate was 0.4 Å/s; pressure was 8 × 10^−7^ Torr. A plate with the samples was rotated at a rate of 20 rotations per minute. The nominal thickness of the silver layer was 60 Å for the substrates without the chromium layer. The substrates with two metals were sprayed sequentially. Firstly, the chromium layer with a nominal thickness of 10 Å was formed. Then, the silver layer with a nominal thickness of 60 Å was created. The substrates were heated at 120 °C for 6 min using the HP-20D-Set thermostat (Daihan Scientific, Wonju, Republic of Korea).

### 2.5. Scanning Electron Microscopy

The surface morphology of the SERS substrates was studied with scanning electron microscopy using the electron microscope Supra 50VP (Zeiss, Oberkochen, Germany) with a resolution of 1.5 nm.

### 2.6. Spectroscopy of Surface Plasmon Resonance

The spectra were acquired using an optical spectrometer for measurement of plasmonic absorption, RamanLife WL (Terasense Group, Inc., San Jose, CA, USA). Prior to the measurement, the spectra were normalized for the silica oxide.

### 2.7. Atomic Force Microscopy

The topology of the surface was studied with a FemtoScan atomic force microscope (Advanced Technologies Center, Moscow, Russia) in the resonant mode. High-resolution sharp silicon cantilevers fpn11 (resonant frequency about 150 kHz, mechanical rigidity of 5.3 N/m) and HA_HR (450 kHz and 34 N/m, respectively) were used. The line frequency was fixed at 0.5 Hz. The FemtoScan Online software (Advanced Technologies Center, Moscow, Russia) was used for signal processing and imaging.

### 2.8. Initial Setup of Aptasensor

SERS substrates were sequentially placed into 1.5 mL-microcentrifuge tubes with the following reagents:

(1) Immobilization of primary aptamers: The substrate was incubated in a 20 nM solution of RHA0385-SH aptamer in water for 5 min.

(2) Surface ‘blocking’ with 1 μM DTT for 2 min.

(3a) Virus binding to the experimental zone: The substrate was incubated for 2 min in IAV diluted with PBS.

(3b) Non-specific binding of allantoic fluid components, or NDV, to the control zone: The substrate was incubated for 2 min with NDV diluted with PBS.

(4) Staining with secondary aptamer: 100 nM or 1 μM RHA0385-Cy3 aptamer in PBS. The time of incubation was 2 min.

(5) Removal of salts with ultrapure water: The time of incubation was 1 min. The total duration was 12 min.

Then, the substrates were placed horizontally, dried in air, and subjected to SERS signal measurement. The spectra were acquired with Enspectr R532 (Enhanced Spectrometry, Meridian, ID, USA) with an exposure time of 2000 ms. The signal was averaged over 10 repeats.

### 2.9. Initial Setup of Aptasensor with Displacement of the Buffer

SERS substrates were sequentially placed into 1.5 mL-microcentrifuge tubes with the following reagents:

(1) Immobilization of primary aptamers: The substrate was incubated in a 20 nM solution of RHA0385-SH aptamer in water for 5 min.

(2) Surface ‘blocking’ with 1 μM DTT for 2 min.

(3a) Virus binding to the experimental zone: The substrate was incubated for 2 min in influenza A virus is diluted with TrisK or TrisNa buffer. 

(3b) Non-specific binding of allantoic fluid components or Newcastle disease virus to the control zone. The substrate was incubated for 2 min with Newcastle disease virus diluted with TrisK or TrisNa buffer.

(4) Staining with a secondary aptamer: 1 μM RHA0385-Cy3 aptamer in TrisK or TrisNa buffer: The time of incubation was 2 min.

(5) Removal of salts with ultrapure water: The time of incubation was 1 min.

The total duration was 12 min.

Then, the substrates were placed horizontally, dried in air, and subjected to SERS signal measurement. The spectra were acquired with Enspectr R532 (Enhanced Spectrometry, Meridian, ID, USA) with an exposure time of 400 ms. The signal was averaged over 20 repeats.

### 2.10. Novel Setup of the Aptasensor

SERS substrates were sequentially modified using the following steps:

(1) Immobilization of the primary aptamer: The substrate was incubated in 1 mL of a 20 nM solution of RHA0385-SH aptamer in TrisNa for 15 min.

(2) Surface washing in 1 mL of ultrapure water for 2 min.

(3a) Virus binding to the experimental zone: The experimental zone of the substrate was incubated in 20 μL of influenza A virus was diluted with TrisNa buffer. The time of incubation was 2 min.

(3b) Non-specific binding to the control zone: The control zone of the substrate was incubated in 20 μL of allantoic fluid or Newcastle disease virus diluted with TrisNa buffer. The time of incubation was 2 min.

(4) Staining with secondary aptamer: 200 nM RHA0385-Cy3 aptamer in TrisNa buffer. The time of incubation was 2 min.

(5) Removal of salts with ultrapure water: The time of incubation was 2 min.

The total duration was 25 min.

Then, the substrates were subjected to SERS signal measurements. The spectra were acquired with Enspectr R532 (Enhanced Spectrometry, Meridian, ID, USA) with an exposure time of 400 ms. The signal was averaged over 30 repeats. Standard deviations were estimated from three independent experiments.

## 3. Results

### 3.1. IAV Distorts the Nanostructured Surface of the Sensor

**Figure 1:**
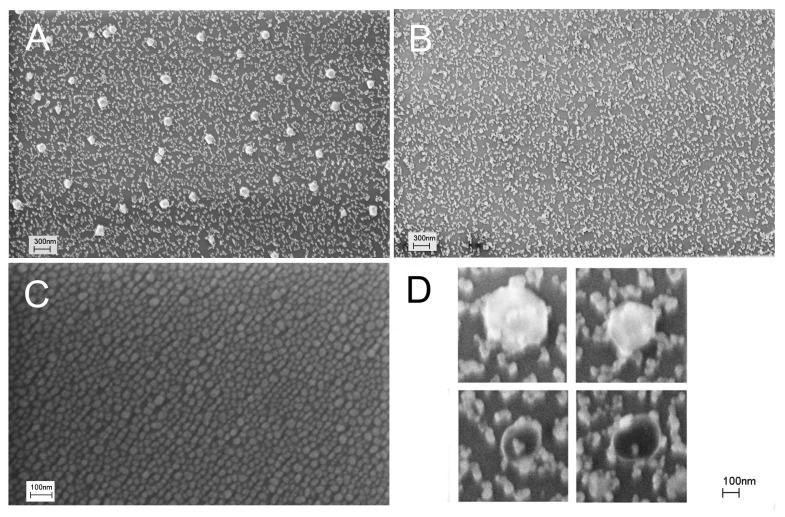
Distortion of the sensor surface during IAV binding was studied with scanning electron microscopy. An initial setup of aptasensors with all steps in PBS is shown (**A**,**B**). A subset A corresponds to a zone treated with IAV (target virus); a subset B corresponds to a zone treated with NDV (off-target virus). Untreated nanostructured surfaces are shown in a subset (**C**). Enlarged images of influenza A virions surrounded by silver nanoparticles are shown in a subset (**D**).

Firstly, we checked whether distortion of the nanostructured surface occurs during virus binding, similar to what was reported in references [22,23]. We used a concentration of IAV 10 times lower than the LoD of the reported sensor, namely, 6 × 10^4^ VP/mL. Scanning electron microscopy revealed large particles in the sensor zones treated with IAV (Figure 1A). The mean diameter of the particles was 110 ± 20 nm, which is close to the expected diameter of IAV of 80–120 nm [29,30]. Electron microscopy was performed without the deposition of additional contrasting agents like heavy metals. Viruses are not to be observed under these conditions, so the most possible explanation for the observed 100 nm nanoparticles is the aggregation of silver nanoparticles around virions. This hypothesis is supported by the large empty spots around the viruses. The enlarged images of the spherical objects are provided in Figure 1D, showing viral particles surrounded by silver nanoparticles. 

The nanostructured surface was significantly distorted by IAV compared to the untreated substrate (Figure 1C). NDV, an off-target virus with a similar size to IAV, was used in the control experiments at a concentration of 4 × 10^5^ VP/mL. The surface did not contain large spherical objects (Figure 1B), but it was also significantly distorted compared to the untreated SERS substrate (Figure 1C). Nanoparticles were merged together due to some suboptimal procedures during the sensor assembly.

**Figure 2:**
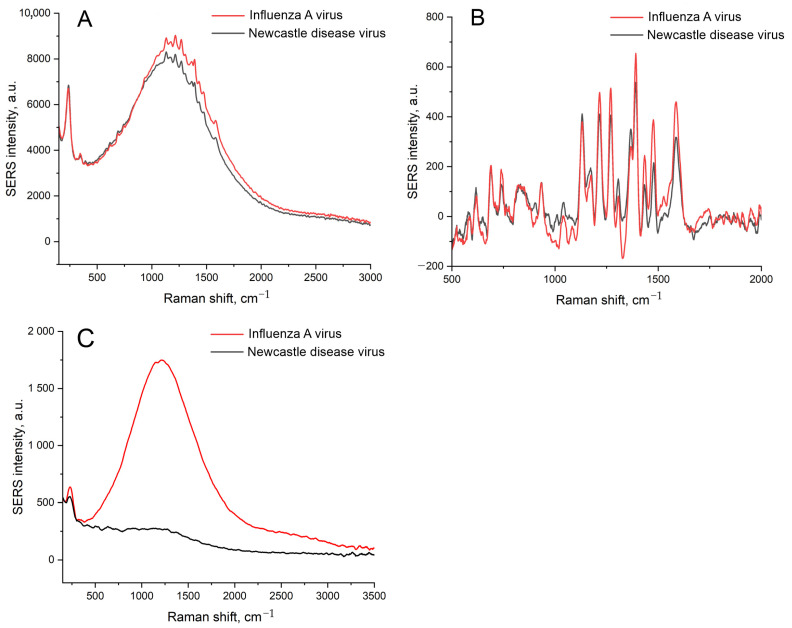
A comparison of the SEL (**A**,**C**) and SERS (**B**) spectra of the sensors with the initial setup with IAV or NDV. A concentration of labeled aptamer was 1 μM (**A**,**B**) or 100 nM (**C**). A concentration of IAV, 6 × 10^4^ VP/mL, was 10-times lower than the LoD of the sensor.

SERS spectra for these samples contained no Raman peaks along with surface-enhanced luminescence (SEL) in the case of a 100 nM concentration of labeled aptamer (RHA0385-Cy3). The increase in the aptamer concentration provided SERS spectra of low intensity with a slight prevalence of intensity in the case of a sample with IAV (Figure 2); these differences were statistically insignificant. The robustness of the sensor could be improved by an empirical selection of the conditions. However, we aimed to reduce the distortion of the surface, as this event is undesirable, providing low reproducibility of the SERS signal. Possibly, the uncontrolled distortion of the nanostructured layer provided non-monotonous dependence on virus content in the previous work [25].

### 3.2. The Distortion of the Nanostructured Surface Is Caused by the Buffer Solution

**Figure 3:**
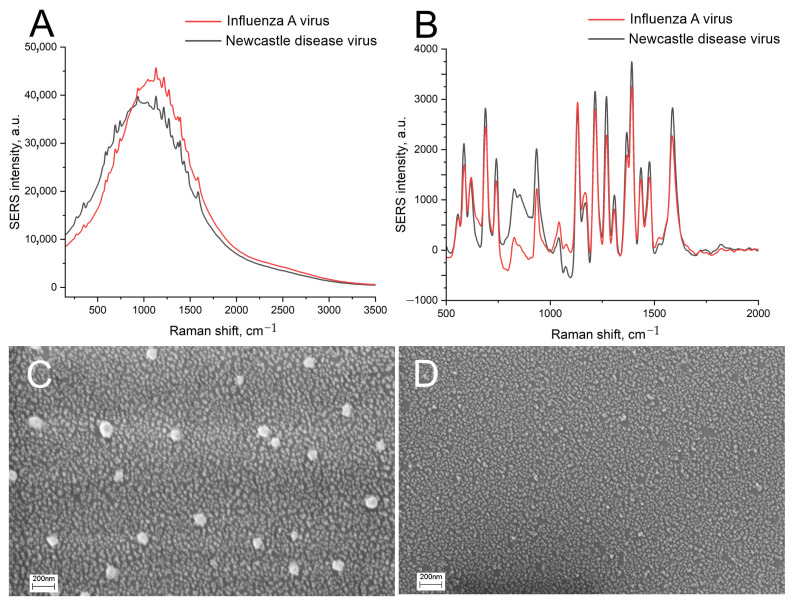
The displacement of the buffer drastically affects the sensor. Comparison of SEL (**A**) and SERS (**B**) spectra of the sensors with the initial setup with displacement of PBS with nitrate-based TrisK buffer. Scanning electron microscopy images of the sensors with the target virus, IAV (**C**), and the off-target virus, NDV (**D**).

The distortion of the nanostructured surface is possibly caused by the biological fluids or PBS buffer used for the dilutions of the labeled aptamers and viruses. PBS buffer contains phosphates and chlorides, which are known to form insoluble salts with Ag^+^. The PBS was displaced with TrisK buffer with tris and KNO_3_; other conditions were kept the same, including the concentrations of the viruses, namely, 6 × 10^4^ VP/mL of IAV and 4 × 10^5^ VP/mL of NDV. The SEL and SERS signals were increased by 5 times (Figure 3A,B), with a negligible discrepancy between target and off-target viruses. The nanostructured layer was minimally distorted by the off-target virus, with several empty zones (Figure 3D). However, the target virus still assembled nanoparticles around the virions. Thus, the substrate nanostructure was unstable in the buffer systems used previously. Interactions between the aptamer and the virus are stronger than the adhesion of the nanoparticles to silica oxide. Further, we used solutions of Tris-buffered sodium/potassium nitrate and optimized the adhesion of the silver nanoparticles as well as the aptasensor setup.

### 3.3. Optimization of Stability of Substrate Nanostructure

**Figure 4:**
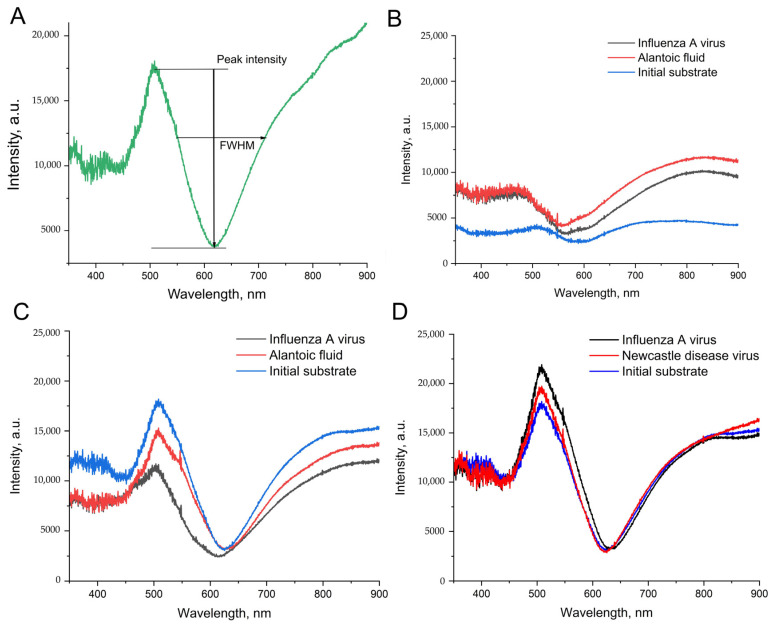
The spectra of surface plasmonic resonance for the nanoisland substrates before and after the treatment with IAV or biological fluid (NDV/allantoic fluid without viruses). (**A**) A peak intensity and a full width at half maximum (FWHM) are the estimated parameters of the spectra. (**B**) The spectra of the substrates without chromium in the setup from ref. [25]. (**C**) The spectra of the substrates with chromium in the setup from ref. [25]. (**D**) The spectra of the substrates with chromium in the novel setup with the displacement of PBS with nitrate-based TrisNa buffer.

The adhesion of silver nanoparticles can be increased with an additional thin layer of chromium [31,32,33]. Surface plasmon resonance (SPR) on a par with scanning electron microscopy was used to estimate the maintenance of the nanostructured layer. Surface plasmon resonance reflects the ability of the nanostructured surface to provide plasmons with the proper characteristics for SERS. The common view of the SPR curve is shown in Figure 4A. The main characteristics are the peak intensity and full width at half maximum (FWHM). The peak intensity correlates with SERS signal enhancement, and FWHM, on par with the wavelength of the negative maximum, correlates with the optimal laser wavelength of the Raman spectrometer. The enhancement factor of the SERS substrate is determined by the following equation: G_SERS_~|A(ω_L_)|^2^|A(ω_S_)|^2^, where A(ω_L_) and A(ω_S_) are amplitude near-field enhancement factors at the laser and Stokes-scattering frequencies, respectively [34]. If the SPR width is large compared to the difference between these frequencies, the enhancement factor is determined by the fourth degree of the amplitude field amplification factors. In our case, the SPR width is not that big and has a sharp profile, meaning that for optimal SERS enhancement and effective input and output Raman resonances, it is crucial to fulfill the following condition: the laser excitation wavelength should be located left to the position of the local minimum of the SPR contour, while the Raman lines near 2000 cm^−1^ should be to the right of the local minimum of the SPR contour.

The silver nanoisland substrates after treatment with PBS and biological fluids have drastically different SPR spectra compared to the untreated sample (Figure 4B). The peak intensities were increased by 2–3-fold, which could be connected with nanoparticle aggregation during the distortion of the surface. These changes are good for SERS intensity. However, the reproducibility of the aggregation is questionable.

Next, the substrates with increased adhesion were prepared. A chromium layer of a width of 1 nm was applied onto the silica oxide layer, and then silver nanoislands were formed onto the chromium layer with heating at the final step. The same setup of the aptasensor provided the opposite results in SPR spectra (Figure 4C). The peak intensities were 1.5–2-fold lower, whereas FWHM was 1.5-fold higher after the treatment with PBS and biological fluids. These differences indicate the distortion of the substrates; the conditions used are suboptimal for biosensor development.

The displacement of the PBS with TrisNa buffer (tris-based buffer with sodium and potassium nitrates) provided much closer SPR curves for the initial substrate and sample substrates after biological fluids (Figure 4D). The related changes in peak intensity and FWHM did not exceed 30%; both parameters were slightly increased in the sample substrates. The displacement of the buffer on a par with the usage of chromium/silver substrates was chosen as a starting point for the further optimization of the sensor’s robustness.

### 3.4. Optimization of the Sensor Setup

**Figure 5:**
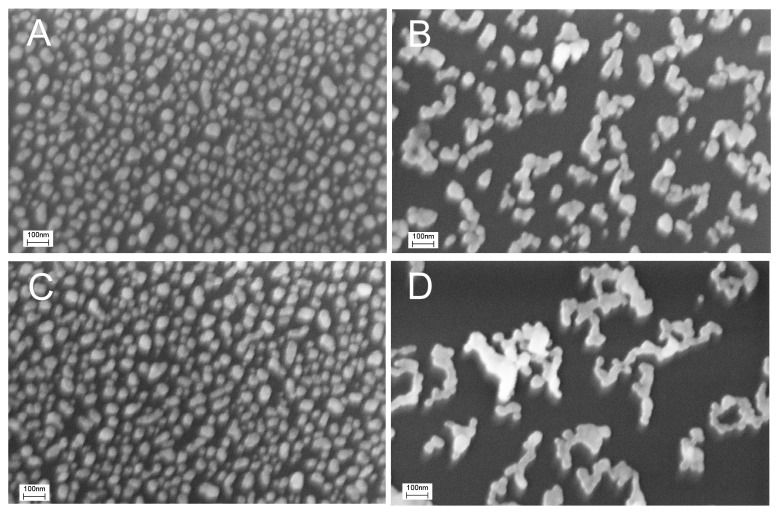
Effect of the buffer and the thiolated aptamer on the distortion of the chromium-treated sensor surface. Scanning electron microscopy images of the untreated sensor (**A**), sensor treated with 200 nM SH-RHA0385-Cy3 aptamer in PBS (**B**) or TrisNa buffer (**C**), and sensor treated with PBS without the aptamer (**D**).

Chromium-treated substrates have nearly the same architecture of nanoislands as substrates without chromium (Figure 5A). The conditions of the aptamer immobilization were optimized. The 200 nM SH-RHA0385-Cy3 aptamer in PBS, TrisNa, or water was incubated for 15 min with the substrates. The aptamer in PBS caused significant distortion of the surface (Figure 5B), whereas the aptamer in TrisNa retained the nanostructured surface nearly intact (Figure 5C). PBS buffer without the aptamer provided even more pronounced aggregation of nanoparticles (Figure 5D). Possibly, the negative charge of the oligonucleotide partially interfered with the aggregation of silver nanoparticles. These results reinforce the critical role of the anion component of the sample in substrate surface stability. Reinforced adhesion of silver nanoparticles did not exclude nanoparticle aggregation in phosphate-chloride solutions.

**Figure 6:**
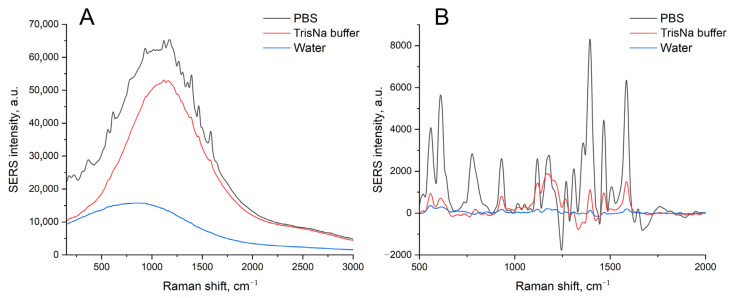
A comparison of the SEL (**A**) and SERS (**B**) spectra of the chromium-treated sensors after immobilization of the labeled aptamer, SH-RHA0385-Cy3, at a concentration of 200 nM in PBS, TrisNa, or water.

The immobilized aptamer, SH-RHA0385-Cy3, was labeled with a Cyanine-3 (Cy3) fluorophore. The samples were subjected to the SERS experiment, which provided the highest intensity of Cy3 SERS spectra for the PBS-treated sample, 5-times lower intensity in TrisNa buffer, and 10-times lower intensity in water (Figure 6). These results can be interpreted in the following way: The ionic strength of the solution is necessary for the efficient immobilization of the aptamer onto the surface, as can be seen from the luminescence intensity (Figure 6A). The most possible reason for the highest SERS in PBS solution is the shift of the peak center of surface plasmon resonance from 628 nm to 535 nm in PBS solution (Table 1). The value of 535 nm coincides with the wavelength of 532 nm of the Raman spectroscope laser.

Previously, the concentration of the aptamer during immobilization was shown to be the critical parameter for sensor maintenance. The best result was obtained in the case of an RHA0385-SH aptamer concentration of 20 nM [25]. This concentration was optimized for the case of the TrisNa buffer used in the initial setup of the aptasensor on the substrates with enhanced adhesion. The largest differences in SEL and SERS for the target (IAV) and off-target viruses (NDV) were observed for the RHA0385-SH aptamer concentration of 20 nM, which suits the previous setup well. The control experiment with off-target oligonucleotides, the RBD-1C-SH aptamer of the SARS-CoV-2 virus, shows no statistically significant differences between IAV and NDV (Table 2). These data proved the specificity of the aptamer toward IAV and the low impact of non-specific binding of IAV to the substrate.

The time of immobilization of the RHA0385-SH aptamer was also optimized. The labeled aptamer, SH-RHA0385-Cy3, was immobilized on the silver nanoislands; SERS intensity was estimated in the time range from 3 to 30 min (Table 3). The maximum SERS intensity was achieved at 15 min. 

The surface of the sensor was blocked with dithiothreitol in the previous setup [25]. Several blocking agents were tested for the new setup, namely, 10, 100, and 1000 nM solutions of dithiothreitol, 6-mercaptohexanol, and spermidine. Thiol-containing blocking agents decreased the SERS signal of the SH-RHA0385-Cy3 aptamer with a simultaneous decrease in the values of SERS(IAV)/SERS(NDV). Spermidine had no effect on the SERS intensity, whereas the value of SERS(IAV)/SERS(NDV) increased with the decrease in spermidine concentration. The most optimal results were obtained when washing with ultrapure water without blocking agents.

### 3.5. Analytical Performance of the New Sensor

**Figure 7:**
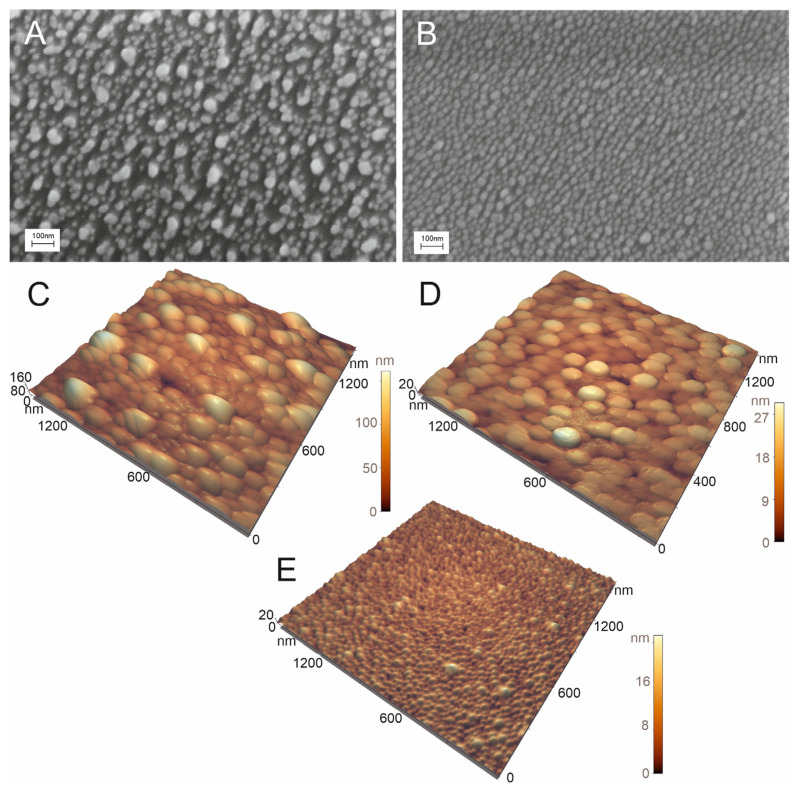
Scanning electron microscopy (**A**,**B**) and atomic force microscopy (**C**–**E**) images of the sensor treated with 6 × 10^4^ VP/mL of IAV (**A**,**C**) or 4 × 10^5^ VP/mL of NDV (**B**,**D**). The untreated substrate is shown in subset (**E**).

The optimized setup of the aptasensor caused minimal, if any, distortion of the nanoisland structure in the control samples (Figure 7B). IAV did cause distortion of the nanoisland structure, but the size of the aggregates was 50 nm, which was twice as low as in the previous setup (Figure 7A). Atomic force spectroscopy revealed large particles with a size of nearly 100 nm in the zone treated with IAV (Figure 7C). Large particles were not found in the zone with NDV disease or the untreated zone (Figure 7D,E). The biological material adsorbed onto the control zone, changing the topology of the substrates according to atomic force microscopy, but virus-like particles were not found. The results of scanning electron microscopy and atomic force microscopy verified the specific binding of IAV to the sensor. Contrary to the previous setup, here the distortion of the nanoisland surface occurred in the case of the target virus only.

**Figure 8:**
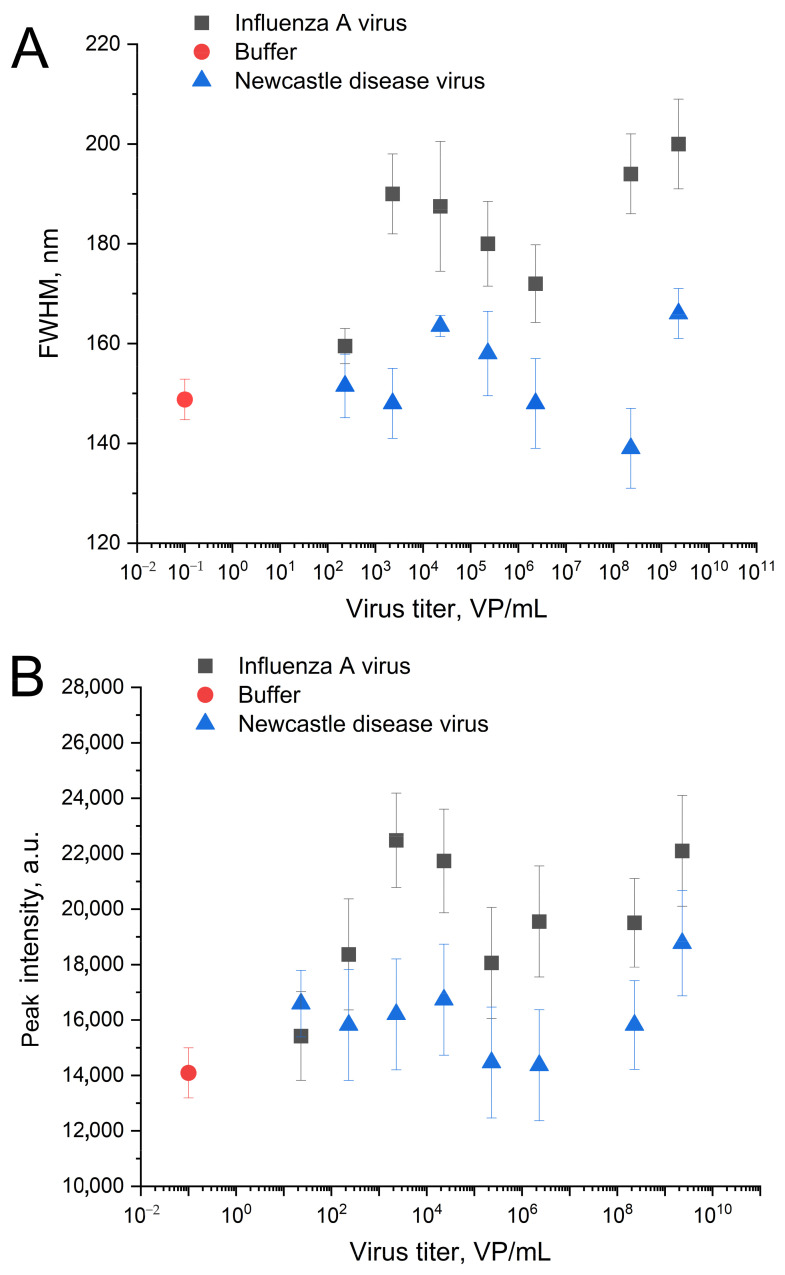
The changes in spectra of surface plasmonic resonance of the nanoisland substrates after the treatment with IAV or NDV in the novel setup. Dependencies of full width at half maximum (FWHM) (**A**) and peak intensities (**B**) on the virus content.

**Figure 9:**
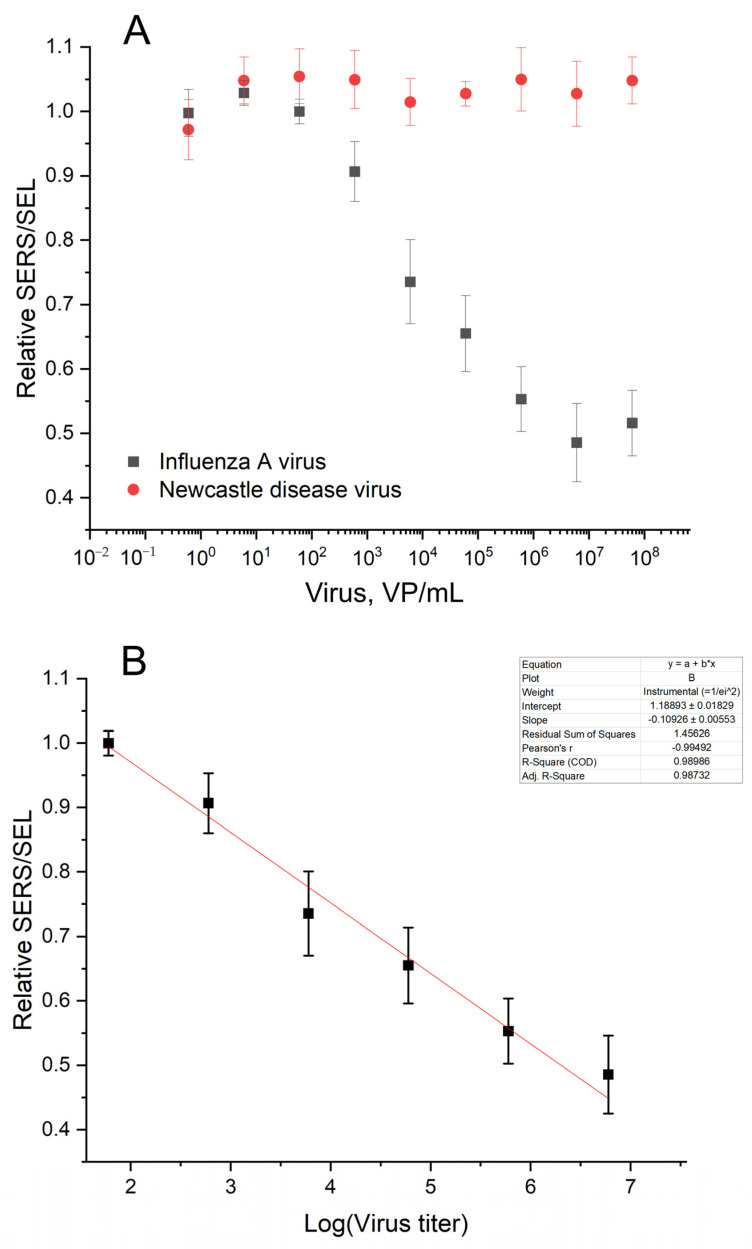
Analytical performance of the aptasensor. Concentration dependence of the aptasensor response in the presence of the target (IAV) and the off-target (NDV) viruses (**A**). The linearization of the dependence for IAV on the logarithmic scale for LoD estimation (**B**).

The IAV-induced distortion of the nanostructured surface caused slight but reproducible changes in the surface plasmon resonance curves of the sensors. Both peak intensity and FWHM values increased in the presence of IAV compared to the zone with off-target virus (Figure 8). The dependence is non-monotonous, which could be attributed to the formation of a monolayer due to the gradual filling of the substrate surface with viruses. Such complexation with the substrate changed the morphology of the nanostructured surface (Figure 7). The optical parameters of the plasmon resonance are particularly sensitive to such changes. Further stratification of the virus only tracks the change in the effective dielectric constant near the silver surface, and therefore the optical properties behave predictably at high concentrations. Slight changes in the presence of off-target viruses are connected with non-specific binding. The aptamer RHA0385 specificity was demonstrated previously [24]; non-specific interactions of metal surfaces with off-targets were accounted for using a normalization procedure (Figure 9).

The SERS and SEL intensities increased in the presence of the influenza A virus. The absolute values had moderate reproducibility from batch to batch of the sensors. However, the relative SERS/SEL ratio calculated as (SERS/SEL)_Experiment zone_/(SERS/SEL)_Control zone_ was highly reproducible. The SERS/SEL ratio was the same in the presence of allantoic fluid and NDV, demonstrating no effect from the off-target virus and a virus-free biological fluid. The relative SERS/SEL ratio provided not only reproducibility but also a monotonous concentration dependence (Figure 9). The relative SERS/SEL ratio decreased monotonously with the increase in IAV content from 60 to 6 × 10^6^ VP/mL. Further increases in IAV did not change the signal reaching the plateau. The limit of detection was estimated as a virus concentration that caused changes greater than three standard deviations from the control sample (allantoic fluid with the same dilution). The LoD was estimated at 190 VP/mL.

## 4. Discussion

Aptasensors provide rapid and sensitive detection of various pathogens [20,35,36,37,38]. Aptamers are compatible with a huge variety of analytical techniques. The development of aptasensors demonstrates an advanced alternative to common laboratory techniques. The strengths of enzyme-free aptasensors include cheap aptamer production compared to recombinant proteins, high stability during storage, and a wide variety of affordable modifications. The main obstacle is the specificity of the aptamers as well as the stability of nucleic acids in biological media. 

SERS aptasensors are among the leading sensors in sensitivity due to the giant enhancement of the signal. Nowadays, portable equipment is available for point-of-care applications. The SERS spectrometer can be as small as 5 cm × 5 cm × 10 cm. The development of affordable and reproducible SERS substrates is another significant issue for the practical implementation of new techniques. Nanoisland substrates are easy to produce because they are not very sensitive to small deviations in the production process due to wide-band plasmon absorption profiles. Unlike SERS substrates with thick layers of metal, the morphology of the nanoisland surface (and, accordingly, the peak of the SPR) could be altered during incubation procedures, which makes it possible to optimally adjust the sensor to the wavelength of laser radiation used. At the same time, the adhesion of the nanostructure layer must be high to retain nanoparticles on the substrate. Fine tuning of nanoparticle adhesion is necessary for reproducible and robust SERS substrates.

Here, we optimized the aptasensor setup to avoid a spontaneous disruption of the nanoisland surface of the SERS substrate. As a result, an aptasensor with a medium LoD and a non-monotonous concentration dependence was transferred into an ultrasensitive aptasensor with the possibility of influenza A virus quantification in a range of 4–5 orders of magnitude (from hundreds to millions of viruses per mL). The accessible LoD of 190 VP/mL is several times lower than the LoD of PCR with reverse transcription [3,4,5]. The time of analysis is substantially lower for the aptasensor (6 min for the aptamer-modified sensor or 25 min for the full setup with aptamer immobilization) compared to PCR with reverse transcription (2–3 h). The reported sensor has the best characteristics among other SERS-based sensors, with the possibility of virus quantification (Table 4). The lithographic nanocolumns [21] are the closest analog in terms of performance, but their cost is significantly higher due to the high-technological equipment and time-consuming production process. This work made a significant step toward affordable SERS-aptasensors for the mass production of tests for influenza A virus. A combination with a portable SERS spectrometer provides the possibility of point-of-care applications outside the laboratory.

## Figures and Tables

**Table 1:** Parameters of surface plasmon resonance spectra of chromium treated silver nanoisland substrates after incubation with the SH-RHA0385-Cy3 aptamer in PBS or TrisNa buffer.

Substrate	Peak Position, nm	Peak Intensity, a.u.	FWHM, nm
Initial	628	5400	150
Aptamer in PBS	535	1100	50
Aptamer in TrisNa	577	6200	130

**Table 2:** Performance of the sensors at different RHA0385-SH concentrations. Chromium-treated silver nanoisland substrates were used. The initial setup was modified using TrisNa buffer instead of PBS. A ratio of the signals from sensors with target (IAV) and off-target (NDV) viruses is provided. The concentrations of viruses were kept at 5 × 10^6^ VP/mL. The off-target aptamer, RBD-1C-SH was used as a control sample. n.d. is not determinable.

RHA0385-SH Concentration, nM	RBD-1C-SH Concentration, nM	SEL(IAV)/SEL(NDV)	SERS(IAV)/SERS(NDV)
2	-	n.d.	1.2 ± 0.1
20	-	2.0 ± 0.2	2.4 ± 0.2
200	-	1.1 ± 0.1	2.2 ± 0.2
-	20	0.9 ± 0.1	0.9 ± 0.1

**Table 3:** Optimization of the time of immobilization of SH-RHA0385-Cy3 aptamer in TrisNa buffer. The aptamer concentration was kept at 20 nM.

Time of Immobilization, min	SERS, a.u.
3	50
5	400
15	1500
30	1100

**Table 4:** Comparison of SERS- or SEF-based sensors for the detection of viral particles with conventional diagnostic assays. N/A—not applicable; RT PCR—reverse transcription polymerase chain reaction; RT LAMP—reverse transcription loop-mediated isothermal amplification. * The recalculation was performed using the ratios from the references [39,40,41]. ** molecular weight of the influenza virus was approximated at 10^7^ Da.

Recognizing Element	Virus Type	Type of the SERS-Substrate	Analytical Performance			Refs
			Limit of Detection	Quantification Range	Time of Analysis	
None	Rheovirus, rinovirus, influenza A, parainfluenza	Gold nanoparticles onto carbon nanotube arrays	10^2^ EID_50_/mL (10^4^ VP/mL) *	No	15 min	[42]
	Respiratory syncytial virus	Silver nanorod array	100 pfu/mL (3 × 10^5^ VP/mL) *	3 × 10^5^–3 × 10^6^ VP/mL	1 h	[43]
	Circovirus, parvovirus, and pseudorabies	Silver nanoparticles onto porous carbon films	1 × 10^7^ VP/mL	No	15 min	[6]
Aptamer	Influenza A	Silver nanoparticles	2.2 × 10^−5^ HAU/mL (10^3^ VP/mL) *	No	15 min	[24]
		Gold nanopopcorn	97 pfu/mL (10^4^ VP/mL) *	10^4^–10^6^ VP/mL	20 min	[44]
		Silver nanoislands onto silica oxide	5 × 10^−4^ HAU/mL (2 × 10^4^ VP/mL) *	No	12 min	[25]
		Silver nanoparticles onto the membrane	10 VP/mL	10–5 × 10^3^ VP/mL	15 min	[22]
		Silver nanoislands onto silica oxide with chromium	190 VP/mL	190–5 × 10^6^ VP/mL	6 min	This work
		Gold nanopopcorn	1.06 HAU/mL (5 × 10^7^ VP/mL) *	5 × 10^7^–5 × 10^9^ VP/mL	15 min	[45]
	SARS-CoV-2		0.95 pfu/mL (7 × 10^5^ VP/mL) *	7 × 10^5^–7 × 10^7^ VP/mL		
		Silver/Chromium/Gold lithographic nanocolumns	100 copies/mL (100 VP/mL)	100–10^7^ VP/mL	15 min	[21]
Antibody	Influenza A	Core-shell nanoparticles loaded with a dye	4 × 10^3^ TCID_50_/mL (4 × 10^5^ VP/mL)	4 × 10^5^–10^9^ VP/mL	3.5 h	[46]
		Gold nanoparticles	30 ng/mL (2 × 10^8^ VP/mL) **	2 × 10^8^–2 × 10^12^ VP/mL	2 h	[47]
	Human immunodeficiency virus	Gold nanostructured surface	350 fg/mL (2 × 10^3^ VP/mL) **	2 × 10^3^–2 × 10^6^ VP/mL	12 h	[48]
RT PCR	Influenza A	N/A	3 × 10^2^–1.2 × 10^3^ VP/mL	Yes, the range is not provided	2–3 h	[4]
	SARS-CoV-2	N/A	1.2 × 10^3^ VP/mL	10^3^–10^7^ VP/mL	2–3 h	[3]
RT LAMP	Influenza A	N/A	10^4^ VP/mL	10^4^–10^7^ VP/mL	1 h	[6]
	SARS-CoV-2	N/A	2 × 10^4^ VP/mL	2 × 10^4^–10^11^ VP/mL	1 h	[7]
Anti-body-based test strip	Influenza A	N/A	1 × 10^6^ VP/mL 20 TCID_50_/mL	No	10–15 min	[1]
	SARS-CoV-2	N/A	7.6 × 10^3^ TCID_50_/mL (5 × 10^8^ VP/mL) *	No	15 min	[2]

## Data Availability

Data are contained within the article.
